# Myeloid Sarcoma: A Rare Case of an Orbital Mass Mimicking Orbital Pseudotumor Requiring Neurosurgical Intervention

**DOI:** 10.1155/2014/395196

**Published:** 2014-11-16

**Authors:** Christopher Payne, William C. Olivero, Bonnie Wang, Seong-Jin Moon, Arash Farahvar, Eric Chen, Huan Wang

**Affiliations:** ^1^School of Medicine, Trinity College Dublin, Dublin, Ireland; ^2^Department of Neurological Surgery, College of Medicine at Urbana-Champaign, Carle Foundation Hospital, University of Illinois, 602 West University Avenue, Urbana, IL 61801, USA; ^3^Department of Internal Medicine, College of Medicine at Urbana-Champaign, University of Illinois, Urbana, IL 61801, USA; ^4^College of Medicine at Urbana-Champaign, University of Illinois, Urbana, IL 61801, USA; ^5^Division of Biology and Medicine, Brown University, Providence, RI 02912, USA

## Abstract

*Objective*. A rare case of myeloid sarcoma (MS), previously referred to as granulocytic sarcoma or chloroma, is presented. Representing a unique form of acute myeloid leukemia (AML), MS may rarely occur in adults. Even rarer, MS may occur as the initial presentation of AML. *Methods*. We report a singular and illustrative case of an orbital pseudotumor mimicking mass in a 65-year-old male as the initial presentation of AML. *Results*. Neurosurgical intervention was required to establish the definitive diagnosis via right modified orbitofrontozygomatic craniotomy as well as to decompress the optic canal, superior orbital fissure, and orbit. *Conclusion*. Postoperatively, he reported decreased pain and improvement of his vision. Further examination revealed decreased proptosis and improved extraocular mobility. Pathological findings demonstrated MS. We review the literature and discuss the neurosurgical relevance of MS as the initial presentation of AML.

## 1. Introduction

Acute myeloid leukemia (AML) is a rare type of leukemia whose frequency increases with age. Myeloid sarcoma (MS) represents a unique form of AML in which a tumor-like proliferation of blast cells occurs outside of the bone marrow [1]. Previously referred to as granulocytic sarcoma and also by the name chloroma meaning “green tumor,” MS has a green appearance due to the presence of myeloperoxidase in many of these tumors [1, 2]. MS more commonly occurs in the pediatric population and in patients with a preexisting diagnosis of AML. Rarely MS may occur in adults. If MS occurs as the initial presentation of AML, diagnosis can be difficult [1, 3–9]. MS has most commonly been reported in skin, bone, and lymph nodes, but it is well accepted that it may occur at almost any location in the body including the orbit [7, 9–11].

## 2. Case Report

**Figure 1:**
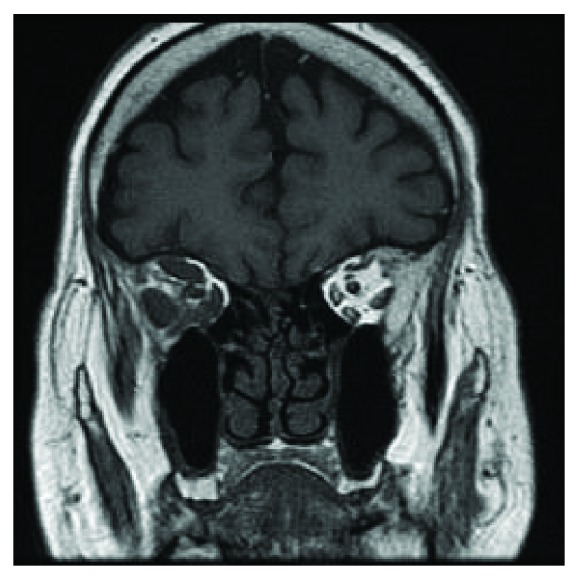
Coronal T1 MRI demonstrating enlargement of all of the extraocular muscles of the right orbit.

**Figure 2:**
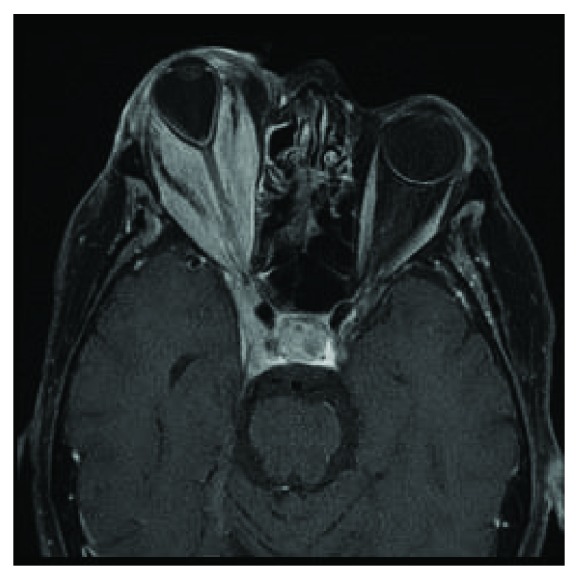
Axial enhanced T1 MRI demonstrating marked proptosis, stretching of the optic nerve, and diffuse enhancement extending into the right cavernous sinus.

**Figure 3:**
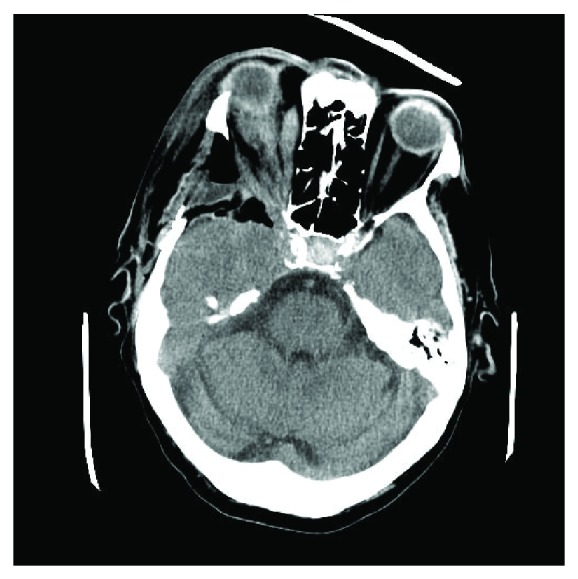
Axial post-operative CT scan demonstrating orbital decompression.

This 65-year-old male presented with a two-month history of right-sided headache, photophobia, double vision, and intermittent eye pain. His presenting visual acuity was 20/50 OD and 20/20 OS. Examination revealed mild proptosis and chemosis of the right eye with restrictive eye movement. CTA demonstrated no evidence of carotid-cavernous fistula. The presence of his cochlear implant made MRI not advisable. Head CT demonstrated right retrobulbar inflammatory stranding associated with mild proptosis and enlargement of the right inferior rectus and medial rectus muscles. Thyroid profile was normal. Peripheral blood work, bone scan, and CT of the chest and neck were also normal. To evaluate for possible infection or malignancy, ophthalmology service performed a needle biopsy. After the needle biopsy was negative for suspicious histology and negative on flow cytometry for lymphoma and leukemia, IOP was tentatively diagnosed and steroids were started. Despite corticosteroids, his clinical condition continued to deteriorate and he soon developed constant holocranial pain, proptosis, chemosis, disc edema, and decreased visual acuity to 20/400 OD without change in the left eye. Because of his rapid clinical decline, he underwent an MRI evaluation despite his cochlear implant. MRI demonstrated a marked increase in size of the mass in comparison to the original CT, as well as findings indicative of extension of the mass into the right cavernous sinus, involvement of all of the extraocular muscles, and marked compression of the globe with stretching of the optic nerve (Figures 1 and 2). He underwent a right modified orbitofrontozygomatic craniotomy to decompress the optic canal, superior orbital fissure, and orbit. Biopsy of the superior rectus muscle was also performed (Figure 3). Postoperatively, he reported decreased pain and improvement of his vision. Further examination revealed decreased proptosis and improved extraocular mobility. Pathological findings demonstrated MS. Bone marrow biopsy and peripheral blood smear were negative but LP demonstrated AML cells. Treatment with 24 Gy of radiotherapy to the brain and eye was carried out followed by chemotherapy with cytarabine and idarubicin including intrathecal chemotherapy via an Ommaya reservoir. However, he ultimately succumbed to the disease.

## 3. Discussion

MS is a rare extramedullary tumorous aggregation of malignant myeloid precursor cells. It usually occurs in the setting of acute myelogenous leukemia, myelodysplastic syndrome, or myeloproliferative disorder. Although first described by Burns in 1811 [12], King in 1853 coined the term chloroma because of the frequent green color [13], later attributed to the enzyme myeloperoxidase [14]. MS can be mistaken on histology for non-Hodgkin's lymphoma, small round cell tumors such as neuroblastoma or undifferentiated carcinoma. Although MS is typically seen in the setting of other myeloproliferative disorders, it can present without a previous history of such disorders. In a series of 21 adult patients with MS, the pleura was the most common location followed by the CNS and skin. The orbit was involved in only 1 patient [15]. In children, however, the orbit is the most common location [16].

Establishing the diagnosis of MS presenting de novo continues to challenge clinicians. In a review of 26 cases of MS, 14 patients presented de novo and all such patients were initially misdiagnosed by the referring pathologist after examination of haematoxylin and eosin (H&E) stained sections. The initial diagnosis was non-Hodgkin's lymphoma in 12 [11]. Even in cases where MS occurs in the setting of previously diagnosed AML, imaging could not differentiate this from other possible complications associated with AML. For this reason, a tissue sample is needed for accurate diagnosis. Appropriate immunohistochemical staining in these cases is particularly essential to establish the diagnosis accurately. Reports have noted a number of reliable markers such as CD43, CD65/KP1, myeloperoxidase, and CD 117. Of these markers, CD43 and CD65/KP1 have been reported in as many as 100% of tissue samples of MS and therefore represent two very reliable markers for identifying this disease [7, 11, 15, 17]. Standard histological staining must be carried out and may suggest a myeloid proliferation; however, spanning the full spectrum of myeloid cell development from poorly differentiated blasts to well differentiated myeloid cells, variation in tissue samples in patients with MS further complicates an accurate diagnosis [1, 11].

Imaging of these tumors is essential to understand the extent and/or spread of this disease; however, this lesion can share features on MRI and CT similar to carcinomas, metastasis, abscess, and lymphoma [6]. On both CT and MRI, the majority of MS cases demonstrate discrete solid masses [6, 16] or involve the lateral rectus muscle [4]. Our patient was unique in that the initial imaging demonstrated involvement of several of the EOMs and then progressed to involve all of the EOMs but with no well-defined mass. Such imaging findings are frequently seen in patients with endocrine orbitopathy or orbital pseudotumor [18–22].

Idiopathic orbital pseudotumor (IOP) is a benign, noninfective inflammatory disorder of the orbit with no known cause. Accounting for the majority of cases of painful orbital mass in adults, it is the third most common orbital disease after thyroid orbitopathy and lymphoproliferative disorders [20]. IOP accounts for approximately 10 percent of all orbital masses. IOP may present acutely, subacutely, or chronically. In contrast with thyroid disease which typically involves both orbits, IOP often occurs in one orbit. The imaging findings in IOP can vary widely depending on the clinical presentations. Involvement of all of the extraocular muscles with extension into the cavernous sinus has been well described [20]. This was similar to the MRI findings in our patient. Most cases of IOP respond to steroids but surgical debulking or radiation therapy is sometimes needed for the steroid nonresponders [20, 23].

The diagnosis of orbital MS is usually accomplished by ophthalmological needle biopsy. However, because the initial biopsy in our case was negative and the patient was rapidly losing vision, neurosurgical intervention was warranted. A bony decompression of the orbit, optic nerve, and superior orbital fissure was performed with opening the falciform ligament and periorbita. Although the external appearance of the extraocular muscles appeared normal, further dissection demonstrated significantly abnormal tissue interspersed with normal appearing muscle fibers. This may explain why the initial biopsy was negative.

Neurosurgeons are commonly consulted for orbital masses that are suggestive of meningioma or optic nerve glioma. However, rarely they may be asked to evaluate patients for orbital decompression.

Orbital decompression is usually undertaken in cases of thyroid orbitopathy or acute elevation of orbital pressure due to hematoma or infection [22, 24]. Rarely, IOP that is unresponsive to steroids may need orbital surgery. A variety of surgical approaches have been used for orbital decompression including the pterional approach [24, 25].

Korinth et al. [26] described an extended pterional approach for patients with acute elevation of intraorbital pressure. They documented improvement in proptosis, visual acuity, and eye motility in the majority of their patients. The same group also reported their results using the same approach in patients with severe Grave's ophthalmopathy. Visual acuity rapidly improved from a preoperative average of 0.53 to 0.77. An average reduction of proptosis of 3.79 mm was achieved. Double vision and restricted eye movement were present in 76.2 percent and improved in 63% [26]. Schick and Hassler reported their series of neurosurgical management of orbital inflammations and infections. Fifteen of the 22 patients were ultimately diagnosed with chronic inflammatory pseudotumor. They used a variety of surgical approaches to perform biopsy and decompression including lateral orbitotomy, transconjunctival, and pterional approaches [23].

It remains debatable whether MS confers a poorer prognosis in patients with AML than AML alone. A number of larger case reports, however, have demonstrated survival rates similar to that of AML in both the adult and pediatric populations with 3- and 5-year survivals at 30% and 21%, respectively [3, 7, 27–32]. Regardless of whether it is associated with systemic AML or not, MS is treated with an aggressive chemotherapy regimen in the same fashion as how systemic AML is treated [3, 15, 33]. If MS is causing compromise to vital structures, debulking surgery and/or radiation treatment of MS patients with isolated chloroma has been shown to reduce the incidence of progression to AML and improved survival [3, 6, 34].

In summary, we present a unique case of MS where initial biopsy was negative. Because of the rapidly declining vision and severely progressive proptosis in our patient, orbital decompression along with open biopsy was carried out. Such neurosurgical intervention resulted in prompt clinical improvement and established an accurate diagnosis.

## Abbreviations

MS: Myeloid sarcoma
AML: Acute myeloid leukemia
IOP: Idiopathic orbital pseudotumor
OD: Right eye
OS: Left eye.
